# Modeling of electric field distribution in tissues during electroporation

**DOI:** 10.1186/1475-925X-12-16

**Published:** 2013-02-21

**Authors:** Selma Corovic, Igor Lackovic, Primoz Sustaric, Tomaz Sustar, Tomaz Rodic, Damijan Miklavcic

**Affiliations:** 1 University of Ljubljana, Faculty of Electrical Engineering, Trzaska cesta 25, SI-1000 Ljubljana Slovenia; 2 University of Zagreb, Faculty of Electrical Engineering and Computing, Unska 3, HR-10000, Zagreb, Croatia; 3 C3M, d. o. o., Centre for Computational Continuum Mechanics, Technological Park 21, SI-1000 Ljubljana, Slovenia

**Keywords:** Electroporation, Numerical modeling, Inverse analysis, Electric field distribution, Tissue conductivity, Liver electroporation, Tumor electroporation

## Abstract

**Background:**

Electroporation based therapies and treatments (e.g. electrochemotherapy, gene electrotransfer for gene therapy and DNA vaccination, tissue ablation with irreversible electroporation and transdermal drug delivery) require a precise prediction of the therapy or treatment outcome by a personalized treatment planning procedure. Numerical modeling of local electric field distribution within electroporated tissues has become an important tool in treatment planning procedure in both clinical and experimental settings. Recent studies have reported that the uncertainties in electrical properties (i.e. electric conductivity of the treated tissues and the rate of increase in electric conductivity due to electroporation) predefined in numerical models have large effect on electroporation based therapy and treatment effectiveness. The aim of our study was to investigate whether the increase in electric conductivity of tissues needs to be taken into account when modeling tissue response to the electroporation pulses and how it affects the local electric distribution within electroporated tissues.

**Methods:**

We built 3D numerical models for single tissue (one type of tissue, e.g. liver) and composite tissue (several types of tissues, e.g. subcutaneous tumor). Our computer simulations were performed by using three different modeling approaches that are based on finite element method: inverse analysis, nonlinear parametric and sequential analysis. We compared linear (i.e. tissue conductivity is constant) model and non-linear (i.e. tissue conductivity is electric field dependent) model. By calculating goodness of fit measure we compared the results of our numerical simulations to the results of *in vivo* measurements.

**Results:**

The results of our study show that the nonlinear models (i.e. tissue conductivity is electric field dependent: σ(E)) fit experimental data better than linear models (i.e. tissue conductivity is constant). This was found for both single tissue and composite tissue. Our results of electric field distribution modeling in linear model of composite tissue (i.e. in the subcutaneous tumor model that do not take into account the relationship σ(E)) showed that a very high electric field (above irreversible threshold value) was concentrated only in the stratum corneum while the target tumor tissue was not successfully treated. Furthermore, the calculated volume of the target tumor tissue exposed to the electric field above reversible threshold in the subcutaneous model was zero assuming constant conductivities of each tissue.

Our results also show that the inverse analysis allows for identification of both baseline tissue conductivity (i.e. conductivity of non-electroporated tissue) and tissue conductivity vs. electric field (σ(E)) of electroporated tissue.

**Conclusion:**

Our results of modeling of electric field distribution in tissues during electroporation show that the changes in electrical conductivity due to electroporation need to be taken into account when an electroporation based treatment is planned or investigated. We concluded that the model of electric field distribution that takes into account the increase in electric conductivity due to electroporation yields more precise prediction of successfully electroporated target tissue volume. The findings of our study can significantly contribute to the current development of individualized patient-specific electroporation based treatment planning.

## Background

Electroporation is an increase of cell membrane permeability to molecules that otherwise lack efficient transmembrane transport mechanisms. Due to membrane electroporation cell membrane conductivity is also increased. Electroporation can be achieved if cells are exposed to a sufficiently high electric field induced by application of high voltage pulses (i.e. electroporation pulses) [1-3]. The efficiency of electroporation strongly depends on pulse parameters such as pulse amplitude, pulse duration, number of pulses and repetition frequency [4]. In order to successfully electropermeabilize a cell membrane the induced transmembrane voltage needs to exceed a given value that depends on cell or tissue type (e.g. cell geometry, cell density, cell’s shape and orientation with respect to the electric field) [5-9]. It has been demonstrated that all types of cells (e.g. human, animal, plant and microorganisms) can be effectively electroporated, provided that appropriate electroporation parameters are selected for each of the cell or tissue type [10]. One of the key parameters for effective cell and tissue electroporation is however adequate electric field distribution within the treated sample [11]. The local electric field induces the transmembrane voltage on the cell [12,13], which is in turn correlated with transmembrane flux [3]. A cell or tissue is reversibly electroporated when the local electric field exceeds a reversible threshold value (E_rev_) [14-16]. If the local electric field however exceeds an irreversible threshold (E_irrev_) value the membranes of the exposed cells are disrupted to such an extent that cells fail to recover and they die (i.e. irreversible electroporation) [17-19].

Increase in cell membrane permeability allows for both administration of different molecules into electroporated cells (such as therapeutic drugs or molecular probes, which otherwise can not enter the cytosol) and for extraction of different cell components out from the cells [20,21]. Since, electroporation has been demonstrated to be a versatile method for both *in vitro* and *in vivo* settings it has been successfully applied to various domains such as medicine (e.g. human and veterinary oncology) [22-27], food processing [28] and microbial inactivation in water treatment [29].

Medical electroporation based therapies and treatments such as electrochemotherapy in local tumor control [27,30], tissue ablation method by means of irreversible electroporation [31,32] and gene therapy and DNA based vaccination by means of gene electrotransfer [33,34] are paving their way into clinical practice. In particular, electrochemotherapy has been demonstrated as an efficient tumor treatment as 85% objective response was obtained in treating skin metastasis of various cancers. In 2006 electrochemotherapy standard operating procedures (SOP) have been defined for the treatment of easily accessible cutaneous and subcutaneous tumor nodules of different histologies [35]. The SOP for electrochemotherapy were defined based on external parameters such as amplitude of applied voltage and distance between electrodes based on preclinical results and the results of numerical calculations in 3D models (1300 V/cm and 1000 V/cm for plate and needle electrodes respectively) [13,15]. The efficacy of SOP for easily accessible cutaneous tumors and subcutaneous tumor (depth: less than 2 cm) nodules were later confirmed by numerous clinical studies [30,36-38]. The *in vivo* studies that were based also on numerical simulations further demonstrated that the protocols of cutaneous tumors can be refined and that the electrochemotherapy can be successfully applied to deep-seated tumors based on analysis of local electric field distribution within the tumors [39,40]. In our previous study [41] we demonstrated that better electrochemotherapy outcome of cutaneous tumors can be obtained with increase of contact surface between plate electrodes and treated tissue. Similarly, Ivorra and colleagues [42] proposed the use of conductive gels in order to ‘homogenize’ the electric field within the treated tissue of easily accessible cutaneous tumors. However, for deep-seated tumors and tumors in internal organs, such as for example liver, brain or muscle tissue that are surrounded by other tissues having different electric properties an individualized patient-specific treatment planning is required as the heterogeneity of local electric field distribution within all exposed tissues can not be neglected [43,44]. Namely, several preclinical and clinical studies have shown that deep-seated tumors located in internal organs (breast cancer [27,37], bone metastasis [45], brain [46], liver metastases [40], melanoma metastasis in the muscle [39]) are also treatable. The outcome of the treatment however greatly depends on positioning of the electrodes, on applying of adequate pulses, electrode geometry and electrical properties of the treated tissues. These findings were either predicted or confirmed with numerical simulations, which resulted in development of computer-based treatment planning of both reversible electroporation based therapies (such as electrochemotherapy [43,44,47-49] and gene therapy and vaccination [50]) and irreversible electroporation based therapies [47,51-53]. Development of treatment planning of electroporation based therapies is currently focused on analysis of the shape and position of electrodes used and applied voltage to the electrodes [40,44,54,55]. Numerous studies demonstrated that the conductivity is increased due to membrane electroporation [2,56,57] and that in calculating the local electric field distribution within treated tissue one needs to account for these tissue conductivity increases [9,16,47,58,59]. In preclinical and clinical studies authors however often consider the treated tissues as being linear electric conductors (i.e. with constant tissue conductivities).

The aim of our study was to investigate whether the increase in electric conductivity of tissues needs to be taken into account when modeling tissue response to the electroporation pulses and how it affects the local electric field distribution within electroporated tissues. We also attempted to identify the nature of functional dependency of tissue conductivity on electric field. We therefore compared linear (i.e. tissue conductivity is constant) and non-linear (i.e. tissue conductivity is electric field dependent) model to the experimental data *in vivo*[56]. The study was performed for both single tissue (one type of tissue, e.g. liver) and composite tissue (several types of tissues, e.g. subcutaneous tumor). Our computer simulations were performed by using three different modeling approaches: 1. inverse analysis (IA) [60,61], 2. nonlinear parametric analysis (NPA) [62] and 3. sequential analysis (SA) [58]. The algorithm for IA modelling approach has been previously developed by [60,61] to model different physical phenomena in wide range of research and industry including biomechanics, pharmaceutical industry and space [63]. In this paper we introduced and successfully applied the IA approach for the first time in the field of modelling of biophysical processes that occur during tissue electroporation.

The results of all three modeling approaches are then compared to the results of our previous experimental measurements *in vivo*[56]. The results of our present study show that the nonlinear models fit experimental data better than linear models. We also demonstrate that tissue conductivity as a function of local electric field need to be taken into account when an electroporation based therapy or treatment is planned.

## Materials and methods

In order to investigate the change of electric properties of tissues on the electric field distribution during electroporation we built 3D numerical models of single tissue and composite tissue.

Modeling of electric field distribution in a single tissue during electroporation was carried out in liver tissue. We analyzed experimental data for plate and needle electrodes. Original experiments with plate electrodes were performed in rat liver, while the experiments with needle electrodes were performed in rabbit liver [56].

Modeling of electric field distribution in composite tissue (i.e. tissue composed of different types of tissues) was carried out in cutaneous tumor tissue. *In vivo* experiments were performed in mice with plate electrodes for two different types of cutaneous tumors: B16 and LPB sarcoma within the study by [56].

The high voltage pulses in all *in vivo* experiments were applied to the electrodes according to the electroporation protocol of eight 100 μs long pulses delivered to the tissues with 1 Hz repetition frequency. Within the experiments [56] the real time tissue electroporation control was performed by monitoring and measuring the electric current during the delivery of electric pulses. In order to evaluate the tissue electroporation efficiency during the pulse delivery the ^51^CrEDTA indicator was used for all tissues. The system for the real time tissue electroporation control and ^51^CrEDTA uptake measurements were described in our previous work [56]. The electric current measured during the *in vivo* experiments provides the information about the bulk tissue conductivity changes during the pulse delivery.

In order to compare and evaluate the results of our numerical models to the experimental data we calculated the goodness-of-fit measure R^2^ between numerically calculated and *in vivo* measured electric current I [A] at the end of the last delivered pulse (i.e. the 8-th pulse) to the tissue.

### Numerical modeling

Electric field distribution in the models of tissue exposed to electroporation pulses U [V] can be determined by solving the equation (Eq. 1) for scalar electric potential. Namely, if we neglect the capacitive transient and time course of conductivity increase during the pulse, we may assume that the current density in tissue is divergence free and electric potential satisfies:

(1) $$-\;\nabla \cdot \left(\sigma \cdot \nabla \varphi \right)=\;0$$

where σ and φ represent tissue conductivity [S/m] and electric potential [V], respectively.

Applied voltage (model input) was modeled as Dirichlet’s boundary condition on the contact surface between electrode and tissue geometry. For the model input values we used the amplitudes of the electroporation pulses applied *in vivo *[56]. In order to mathematically separate the conductive segment from its surroundings we applied Neuman’s boundary condition (J_n_ = 0, where J_n_ is the normal electric current density [A/m^2^]) on all outer boundaries of our models.

As long as the applied voltage U [V] is low enough the tissue conductivity σ [S/m] can be treated as a constant (i.e. σ = const. in Eq. 1), the problem is therefore described by the Laplace’s equation which is a linear partial differential equation that can be easily solved numerically. In this case the tissues can be modeled as a linear conductor with linear current–voltage I(U) relationships due to the constant tissue conductivities since the amplitude of the applied electroporation pulses is too low to produce an E above the reversible electroporation threshold (E < E_rev_).

However, to account for experimentally observed tissue conductivity increase due to electroporation, Eq. 1 becomes nonlinear partial equation as σ is no longer constant. If the local electric field in the tissues exceeds the E_rev_ value, electric properties of treated tissue change (i.e. tissue conductivity increases due to the electroporation process). In this case the σ in Eq.1 depends on the electric field intensity E: σ = σ(E). Namely, during the application of electroporation pulses, tissue conductivity increases according to the functional dependency of the tissue conductivity on the local electric field distribution σ(E), which in our study describes the dynamics of the electroporation process. This subsequently results in nonlinear electric current voltage relationship I(U) (i.e. numerical model of tissue is non-linear).

Measurement of electric current I [A] during delivery of eight rectangular electroporation pulses of 100 μs duration and 1 Hz repetition showed that after rapid capacitive transient (which is due to tissue capacitance) the current increased towards the end of the pulse [56,52]. The rate of increase of electric current I [A] depends on the applied voltage U [V], which provides information about the changes in electric properties of the treated tissue (i.e. changes in electric conductivity of the tissue σ [S/m]). The rates of increase of I and σ thus allow us to identify the degree of tissue electroporation.

Numerical calculations of Eq.1 and tissue electroporation dynamics σ(E) in our study were performed with three different approaches that were based on finite element method: Inverse analysis (IA), nonlinear parametric analysis (NPA) and sequential analysis (SA). The software for IA was previously developed by Center for Computational Continuum Mechanics (Ljubljana, Slovenia) [61], while for the NPA and the SA we used Comsol Multiphysics software (version 3.5a, COMSOL, Sweden).

The three abovementioned modeling approaches are described here below:

#### Inverse analysis (IA)

The IA is based on standard Newton–Raphson method, which is widely used for calculating nonlinear problems based on finite element method. The tissue electroporation dynamics i.e. the dependence of the electrical conductivity with respect to electrical field strength σ(E) is described by linear and exponential relationships. Unknown parameters of these relationships are calculated by IA [60]. The IA is performed by using a symbolic system AceGen (Multi-language, Multi-environment Numerical Code Generation) [64] with AceFeM environment (The Mathematica Finite Element Environment) [65] for development of finite element equations and generation of corresponding finite element user subroutines. This enables symbolic formulation of the problem at a high abstract level. The derivation presented here follows a general approach to the automation of primal and sensitivity analysis of transient problems introduced by [66]. Below is a brief description of the formulation.

The response of the steady-state non-linear problem is defined by the global residual vector *R* and the vector of unknown variables of the problem *φ* (Eq. 2):

(2) $$R\left(\varphi \right)=*\sum_{e}R_{e}\left(\varphi \right)=0$$

where *R*_*e *_represents the contribution of the *e-*th element to the global residual and *e* is the element number. The operator *Σ is chosen here instead of the ordinary *Σ* symbol in order to denote that an assembly process takes place in which all element contributions have not only to be added up but also the kinematical compatibility between the elements has to be fulfilled [60]. The residual vector *R* is a part of standard notation used in finite element analysis. The finite element problems are represented by a set of equations in the residual form, which are solved iteratively using the Newton–Raphson method. The system given by the Eq. 2 can therefore be solved by the Newton–Raphson method, in which the following iteration is performed according to Eq. 3 and Eq. 4:

(3) $$\frac{\mathrm{dR}}{\mathrm{d\varphi}}\left(\varphi^{\left(i\right)}\right)\;\Delta \varphi =-R\left(\varphi^{\left(i\right)}\right)$$

(4) $$\varphi^{\left(i+1\right)}=\varphi^{\left(i\right)}+\Delta \varphi$$

The terms *R*(*φ*^(*i*)^) and $\frac{\mathrm{dR}}{\mathrm{d\varphi}}\left(\varphi^{\left(i\right)}\right)$ are referred to as the residual (or load) vector and the tangent operator (or tangential stiffness matrix), respectively. The independent solution vector *φ*^(*i*)^ is updated after the linear system given by the Eq. 3 has been solved for the unknown increment *Δφ*. The upper index ^(*i*)^ refers to the quantities at the *i-*th iteration step of the outer loop.

The distribution of static electric field is determined by solving the Eq. 1. By using integral form and Galerkin approximation one can obtain

(5) $$\int_{V}\nabla \delta \varphi .\sigma .\nabla \varphi \;\mathrm{dV}-\;\int_{S}J.n\;\delta \varphi \;\mathrm{dS}=0$$

where *φ* is the electric potential, *V* is volume, *S* is surface and *J* is current density.

Discretization of Eq. 5 by *φ* = *N*_*e*_. *φ*_*e*_ and *δφ* = *N*_*e*_. *δφ*_*e*_ = *N*_*e*_ leads to the Eq. 6:

(6) $$R_{e}\left(\varphi \right)=\left[\int_{V_{e}}\nabla N_{e}.\sigma .{\left(\nabla N_{e}\right)}^{T}\;\mathrm{dV}\right].\varphi_{e}$$

where *φ* is the electric potential, *φ*_*e*_ are the electrical potentials in the element nodes, *δφ* is the test function, *N*_*e *_are the nodal shape functions and σ is the electrical conductivity.

We considered linear (Eq. 7) and exponential (Eq. 8) dependences of tissue conductivity *σ* with respect to electric field strength *E* as follows:

(7) $$\sigma \left(E\right)=\left(\sigma_{0}+k_{\sigma }\;E\right)\;I$$

(8) $$\sigma \left(E\right)=\left(\sigma_{0}+c_{1}\;\left(e^{c_{2}\;E}-1\right)\;\right)I$$

where electric field strength *E* is defined as:

(9) $$\begin{array}{l} E=-\nabla \varphi \\ E=\sqrt{E.E} \end{array}$$

and **I** is the identity matrix. The constant in Eq. 7 and Eq. 8 stands for the baseline tissue conductivity of non-electroporated tissue (i.e. σ_0_ = σ(0)), while the constants *k*_*σ*_, *c*_1_ and *c*_2_ stand for the parameters of linear (Eq. 7) and exponential (Eq. 8) functions describing electroporated tissue (i.e.σ(E)).

Unknown coefficients *σ*_0_, *k*_*σ*_, *c*_1_ and *c*_2_ were determined with IA [60] by minimizing the objective function *f* of the following form:

(10) $$f\left(\sigma_{0},k_{\sigma },c_{1},c_{2}\right)=\sum_{j}{\left(\frac{I_{\mathrm{FEM}}-I_{\text{measured}}}{U}\right)}_{\left(j\right)}^{2}$$

Determination of unknown coefficients *σ*_0_, *k*_*σ*_, *c*_1_ and *c*_2_ allowed us to detect the properties of the treated tissue for both situations σ = const. (i.e. σ_0_ = σ(0)) and σ(E)).

By using the Newton's method in the Mathematica (version 8.0) programming environment the minimization problem is defined as:

(11) $$g\left(x=\left\{\sigma_{0},k_{\sigma },c_{1},c_{2}\right\}\right)=\left\{\begin{array}{l} \frac{\partial f\left(\sigma_{0},k_{\sigma },c_{1},c_{2}\right)}{\partial \sigma_{0}}=\sum_{j}{\left(2\;\frac{I_{\mathrm{FEM}}-I_{\text{measured}}}{U^{2}}\;\frac{\partial I_{\mathrm{FEM}}}{\partial \sigma_{0}}\right)}_{\left(j\right)},\\ \frac{\partial f\left(\sigma_{0},k_{\sigma },c_{1},c_{2}\right)}{\partial k_{\sigma }}=\sum_{j}{\left(2\;\frac{I_{\mathrm{FEM}}-I_{\text{measured}}}{U^{2}}\;\frac{\partial I_{\mathrm{FEM}}}{\partial k_{\sigma }}\right)}_{\left(j\right)}\\ \frac{\partial f\left(\sigma_{0},k_{\sigma },c_{1},c_{2}\right)}{\partial c_{1}}=\sum_{j}{\left(2\;\frac{I_{\mathrm{FEM}}-I_{\text{measured}}}{U^{2}}\;\frac{\partial I_{\mathrm{FEM}}}{\partial c_{1}}\right)}_{\left(j\right)}\\ \frac{\partial f\left(\sigma_{0},k_{\sigma },c_{1},c_{2}\right)}{\partial c_{2}}=\sum_{j}{\left(2\;\frac{I_{\mathrm{FEM}}-I_{\text{measured}}}{U^{2}}\;\frac{\partial I_{\mathrm{FEM}}}{\partial c_{2}}\right)}_{\left(j\right)} \end{array}\right\}=0$$

where *U* is the voltage between the electrodes, *I*_*FEM*_ is the calculated current and *I*_*measured *_the measured current in the *j*-th measurement. The derivatives $\frac{\partial I_{\mathrm{FEM}}}{\partial \sigma_{0}}$, $\frac{\partial I_{\mathrm{FEM}}}{\partial k_{\sigma }}$, $\frac{\partial I_{\mathrm{FEM}}}{\partial c_{1}}$ and $\frac{\partial I_{\mathrm{FEM}}}{\partial c_{2}}$ were calculated within the finite element method analysis.

#### Nonlinear parametric analysis (NPA)

The NPA has been developed in our previous study [62]. In the present study we used NPA to solve the equation Eq. 1 for both conditions σ = const. (for E < E_rev_) and σ = σ(E) (for E ≥ E_rev_) by using Comsol Multiphysics’ stationary nonlinear solver. Namely, we treat the problem as stationary since we analyzed the tissue behavior by calculating the σ(E) dependency at the end of the applied pulse U [V]. The parameters defined by NPA are the values of the applied voltages U (i.e. model inputs). The electric current I [A] is calculated as a model output providing the value of I [A] at the end of pulse. The Comsol Multiphysics’ nonlinear solver uses an affine invariant form of damped Newton method. The user must supply an initial guess for the dependent variable U (i.e. U_0_). Starting with the initial guess, the software forms the linearized model using U_0_ as the linearization point i.e. the discrete form of the equations as f(U) = 0, where f(U) is the residual vector and U is the solution vector. The iterative Newton method can be applied in the usual way, expanding the residual vector in a Taylor series, neglecting all but the first order term. The software solves the discretized form of the linearized model f ’(U_0_)δU = −f(U_0_) for the Newton step δU by using the selected linear system solver (the f ’(U_0_) is the Jacobian matrix). It then computes the new iterate U_1_ = U_0_ + λδU, where λ is the damping factor. The modified Newton correction then estimates the error E_error_ for the new iterate U_1_ by solving f’(U_0_)E_error_ = −f(U_1_). If the relative error corresponding to E is larger than the relative error in the previous iteration, the code reduces the damping factor λ and recomputes U_1_. This algorithm repeats the damping-factor reduction until the relative error is less than in the previous iteration or until the damping factor underflows the minimum damping factor. When it has taken a successful step U_1_, the algorithm proceeds with the next Newton iteration. A value of λ = 1 results in Newton’s method, which converges quadratically if the initial guess U_0_ is sufficiently close to the solution. Comsol Multiphysics will reduce the damping factor and re-compute U_1_ if the relative error is larger than the previous value. The purpose of this damping factor is to make the process converge for a broader range of initial values (initial guesses U_0_).

It is necessary to increase the voltage U from 0 V in small steps and use the solution from a previous step as the initial condition for the next step. This is taken care of automatically by the parametric solver. The parametric solver consists of a loop around the stationary solver. If the nonlinear solver does not converge it tries a smaller parameter step; if it does converge it determines the size of the next parameter step based on the speed of the convergence of the Newton iteration.

#### Sequential analysis (SA)

The SA in our study was based on the sequential permeabilization model proposed by [58], where changes in tissue conductivity were used as an indicator of tissue permeabilization. Namely, the dynamics of electroporation was modeled as a discrete process with the sequence of static finite element models. Each of the finite element models described the process in one discrete interval (each of the discrete intervals relates to a real, discrete, but undetermined time interval). In each static finite element model in sequence, the tissue conductivity was determined based on the electric field distribution from the previous model in the sequence according to the Eq. 12:

(12) $$\sigma \;\left(k\right)=f\;\left(E\left(k-1\right)\right)$$

where *k* stands for the number of static finite element models in sequence.

Model input is the applied voltage pulse, and model outputs are the electric field distribution E and total electric current I in each specific sequence k. The increase in electrical current I from I_0_ to I_k_ simulates the tissue response during the delivery of the electroporation pulses U in each discrete interval k (static finite element model in sequence) to the tissue electroporation (i.e. due to the functional dependency of σ on the electric field distribution E). If the electroporation does not occur, σ remains constant, thus I = I_0_. More detailed description of SA is given in [16,58,59].

The numerical calculations obtained with NPA and SA by using COMSOL Multiphysics 3.5a software package were performed in the 3D Conductive Media DC application mode on a computer running Windows XP (64 bit) with a 3.00 GHz Intel Pentium D processor and 2 GB of RAM.

For the numerical calculations with IA we used AceGen (version AceFEM 3.101) system [61,64,65]. The calculations were performed on a computer running Windows XP (64 bit) with 2.67 GHz Intel Core i7 CPU and 6 GB of RAM.

Results of numerically calculated model outputs (such as total current and local electric field distribution) with all three approaches were controlled for numerical errors by increasing the mesh density until the output results changed by less that 0.5%. The mesh of the resulting finite element models of liver tissue electroporated with plate and needle electrodes were composed of approximately 30 000 and 25 400 elements, respectively. Number of generated finite elements of the tumor mesh model was 30 268.

### Model geometry and electric properties of a single tissue

**Figure 1:**
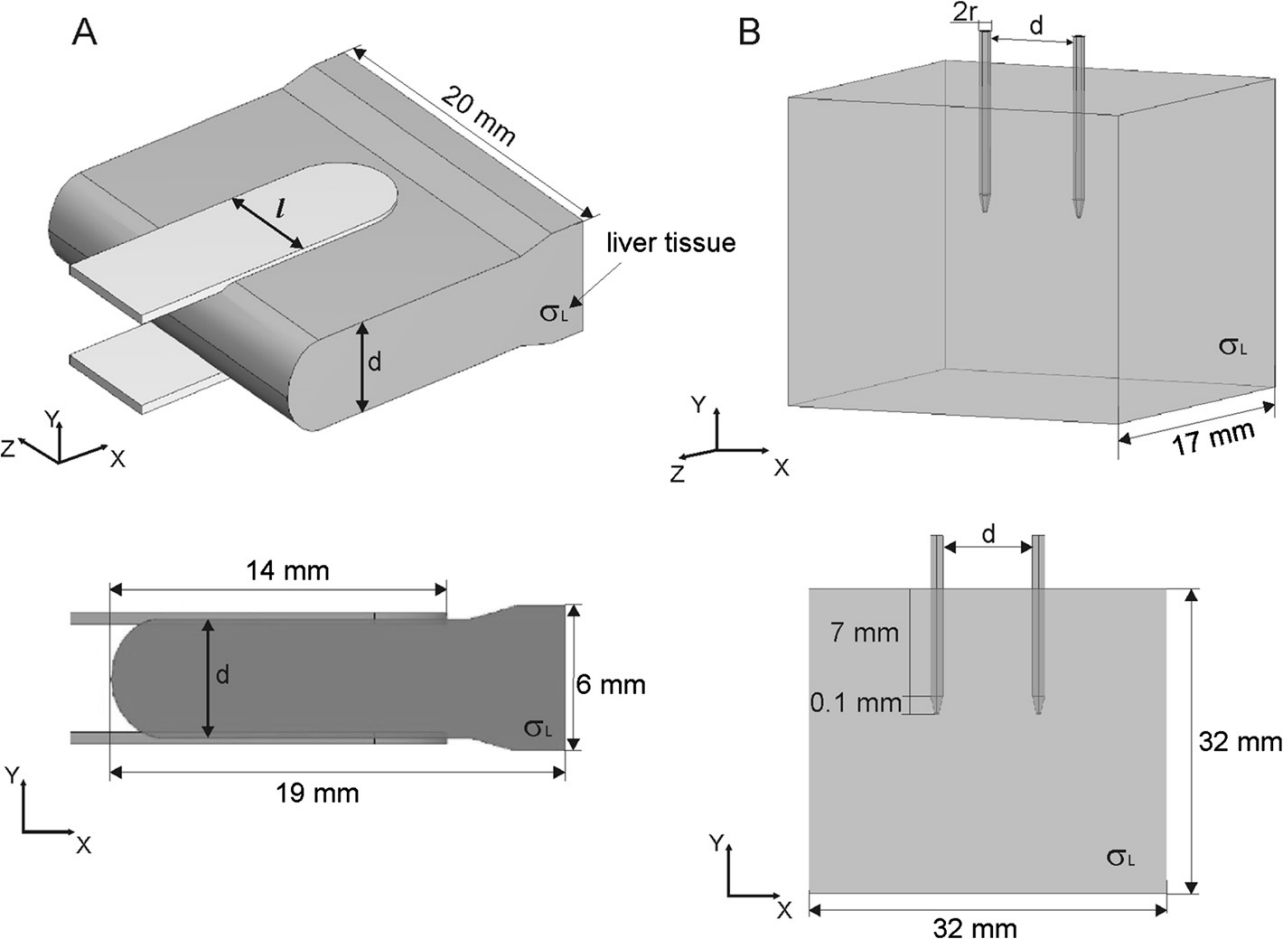
**Geometry of numerical models of liver tissue. A**. treated with plate electrodes and **B**. treated with needle electrodes. The parameter σ_L_ stands for the conductivity σ [S/m] of liver tissue.

The 3D geometry of the numerical model and its 2D cross-sectional view in XY plane of a single tissue (i.e. liver tissue) is given in Figure 1. The model of liver tissue electroporated with plate electrodes is given in Figure 1A, while the model of liver tissue electroporated with a pair of needle electrodes is given in Figure 1B.

By using the IA numerical simulations we identified both baseline electric conductivity of tissue σ_0_ (for the condition σ(0)) and linear and exponential σ(E) (Eqs. 7 and 8) that fitted best the experimental data *in vivo*[56]. The determined σ_0_ and σ(E) for plate and needle electrodes were then compared to the data from the literature.

Since the NPA and SA required the baseline tissue conductivity σ_0_, the conductivity increase factor due to electroporation, the reversible and ireversible threshold values (E_rev_ and E_irrev_) to be predefined, in our models we selected the data from [58,62,67,68]. The functional dependencies σ(E) predefined in the models were sigmoid-like function implemented as smoothed Heaviside's step function [62] with continuous second derivative and sigmoid function σ(E) that was implemented by Sel and colleagues [58].

### Model geometry and electric properties of composite tissue

**Figure 2:**
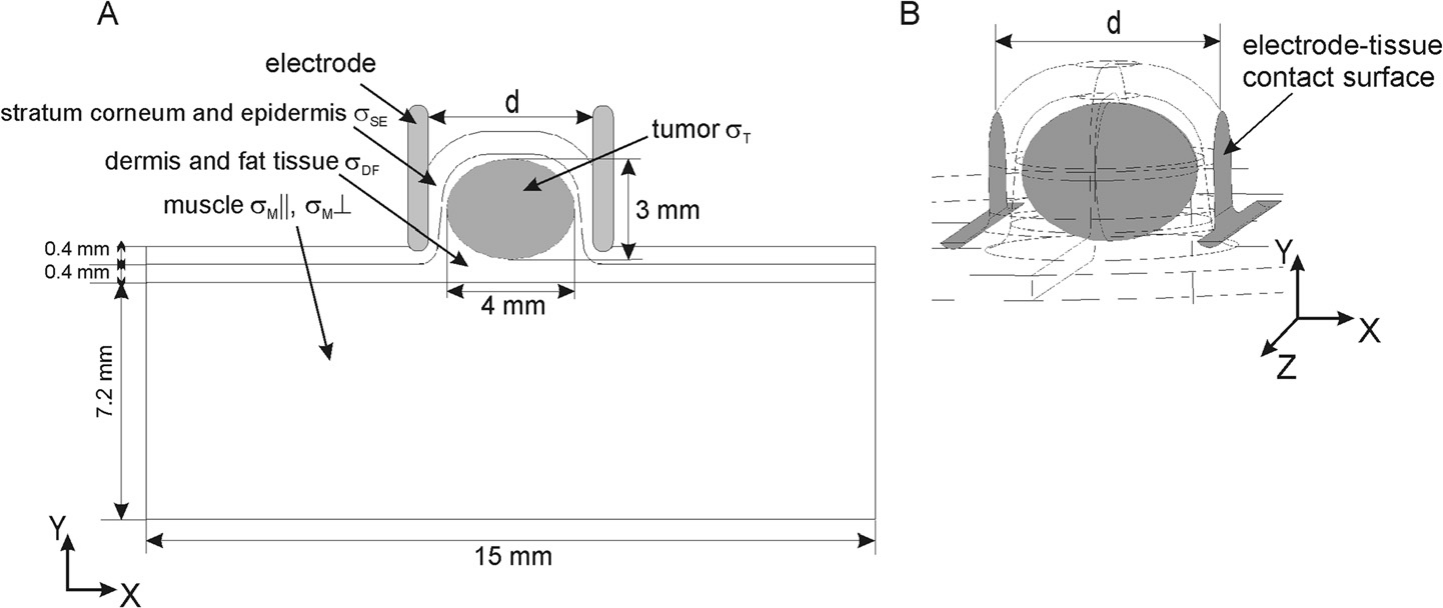
**Geometry of numerical model of subcutaneous tumor. A**. The geometry dimensions and composition of cutaneous tumor model. (The electrodes in this figure only sketch the site of electrodes’ position in the experiments and do not take part of the numerical model) and **B**. 3D geometry of tumor model with the contact surface between tissue and electrodes. The distance between plate electrodes was d = 5.2 mm.

The geometry of composite tissue i.e. subcutaneous tumor is shown in Figure 2A. The distance between two plate electrodes used in experiments was d = 5.2 mm. The tumor geometry was created so as to match as accurate as possible the electrode placement and the geometry of the tissue treated within the *in vivo* experiments. The electrodes in Figure 2A only sketch the site of electrodes’ position in the experiments and do not take part of the numerical model. The body of the electrodes was not modeled in our numerical model in order to reduce the computation time of numerical simulations. The electrodes were modeled rather as boundary conditions defined on the electrode-tissue contact surfaces (Figure 2B).

The numerical model of the tumor was built as a heterogeneous 3D model composed of four types of tissues with different electric properties (i.e. electric conductivities). The skin in our subcutaneous tumor model was modeled as two-layer tissue: the first layer comprised the electric properties of stratum corneum and epidermis (σ_SE_), while the second layer comprised dermis and fat tissue (σ_DF_). The underlying muscle tissue was modeled as anisotropic tissue with σ_M⊥_ (Y and Z direction -Figure 2) and σ_M||_ (X direction - Figure 2) in perpendicular and parallel direction of E with respect to the muscle fibers. The baseline conductivities of the modeled tissues (i.e. the initial σ of non-electroporated tissues for E < E_rev_), the conductivity increase factor due to tissue electroporation and the threshold values E_rev_ and E_irrev_ for each of the tissues were selected based on data we published previously [9,16,59,69]and based on the comparison of I [A] calculated in our models to the *in vivo* measurements. Our numerical and experimental analysis was performed for two different types of cutaneous tumors: B16 melanoma and LPB sarcoma.

The relationship of electric conductivity with respect to the local electric field σ(E) for all modeled tissues was considered to be smoothed Heaviside step function taking into account the electric conductivities and the threshold values data listed in Table 1. For the numerical calculations we used NPA. We examined the electric current and local electric field distribution within the model of subcutaneous tumor for three different electric properties of target tumor tissue σ_T1_, and σ_T2_ and σ_T3_.

**Table 1:** The defined parameters of tissues composing the subcutaneous tumor model

**Tissue**	**E**_**rev **_**[V/cm]**	**E**_**irrev **_**[V/cm]**	**Initial σ for E < E**_**rev**_	**σ increase for E > E**_**irrev **_**vs E < E**_**rev**_
Stratum corneum and epidermis	400	1200	σ_SE_ = 0.008 S/m	100
Dermis and fat	300	1200	σ_DF_ = 0.25 S/m	4
Tumor	400	800	σ_T1_ = 0.3 S/m	2.5
			σ_T2_ = 0.6 S/m	
			σ_T3_ = 0.9 S/m	
Muscle transversal	200	800	σ_M_⊥ = 0.135 S/m	2.5
Muscle longitudinal	80	800	σ_M_|| = 0.75 S/m	2.5

The baseline conductivities σ of modeled tissues, reversible and irreversible threshold values (E_rev_ and E_irrev_) of the tissues and the rate of increase in tissue conductivity due to electroporation. The relationship σ (E) for all tissues was smoothed Heaviside function.

### Statistical analysis

To compare the outputs of our models to the experimentally measured data we calculated coefficient of determination R^2^, which represents a statistical measure of how well the model data approximates the real data points (i.e. the measure of the goodness-of-fit between the modeled and measured data). The R^2^ = 1 means that the model data perfectly fit the real data points. If the R^2^ has a value closer to the 0 or a negative value the model data do not fit the real data points.

The coefficient of determination is given by the Eq.13.

(13) $$R^{2}=1-\frac{SS_{\mathrm{err}}}{SS_{\mathrm{tot}}}$$

where *SS*_*err*_ and *SS*_*tot*_ are residual sum of squares and the total sum of squares, respectively.

The "variability" of the data set is measured through the sums of squares *SS*_*err*_ and *SS*_*tot*_.

The residual sum of squares *SS*_*err*_ is defined by the Eq. 14:

(14) $$SS_{\mathrm{err}}={\sum_{i}\left(y_{i}-f_{i}\right)}^{2}$$

where *y*_*i*_ data stand for the measured values of electric current at the end of the pulse, each of which has an associated modeled value *f*_*i*_.

The total sum of squares SS_tot_, which is proportional to the sample variance, is given by the Eq. 15:

(15) $$SS_{\mathrm{tot}}={\sum_{i}\left(y_{i}-\bar{y}\right)}^{2}$$

where the $\bar{y}$ is the mean of the measured data *y*_*i*_, which is given by the Eq. 16:

(16) $$\bar{y}=\frac{1}{n}\sum_{i}^{n}y_{i}$$

where *n* is the number of observations.

## Results

### Single tissue

#### Plate electrodes

Modeling of electric field distribution during electroporation with plate electrodes was performed with IA and with NPA for the model geometry given in Figure 1A. The geometry details are identical as in *in vivo* experiments [56] and in our previous work where the NPA was first implemented [62].

**Figure 3:**
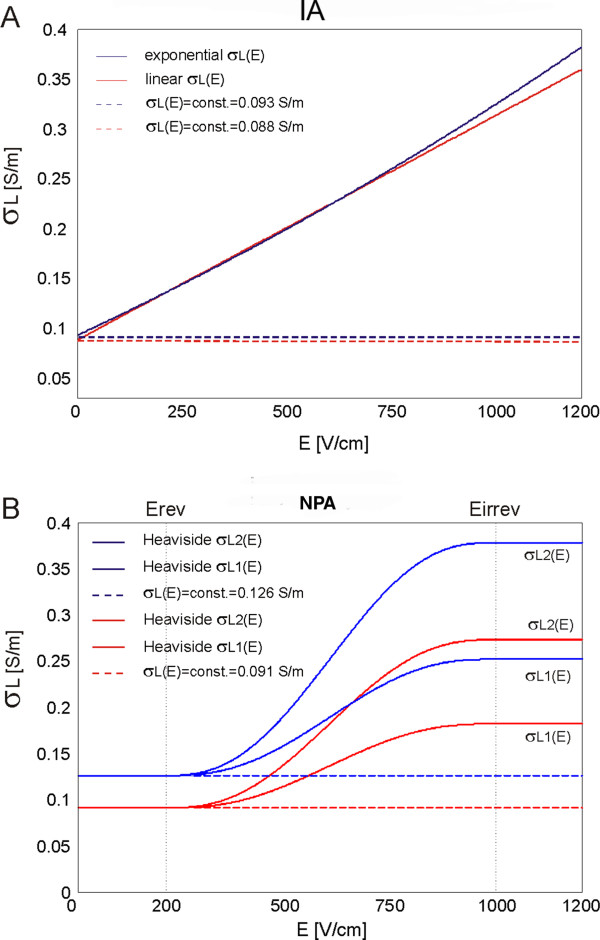
**Baseline conductivities (σ**_**L**_**(E) = σ**_**0**_ **= const.) and tissue conductivity vs. electric field dependencies σ**_**L**_**(E). A**. σ_0_ and σ_L_(E) identified by using IA and **B**. σ_0_ and σ_L_(E) predefined in our numerical models based on the data from literature – smoothed Heaviside σ_L_(E) was predefined for the numerical calculations with NPA. σ_L1_(E) and σ_L2_(E) stand for the smoothed Heaviside relationships σ_L_(E) with conductivity increase factor (due to electroporatoion) of two and three, respectively.

By using IA we first identified linear and exponential relationship σ(E) (Eqs. 7 and 8) and determined corresponding baseline tissue conductivity σ(0) (Figure 3A).

**Figure 4:**
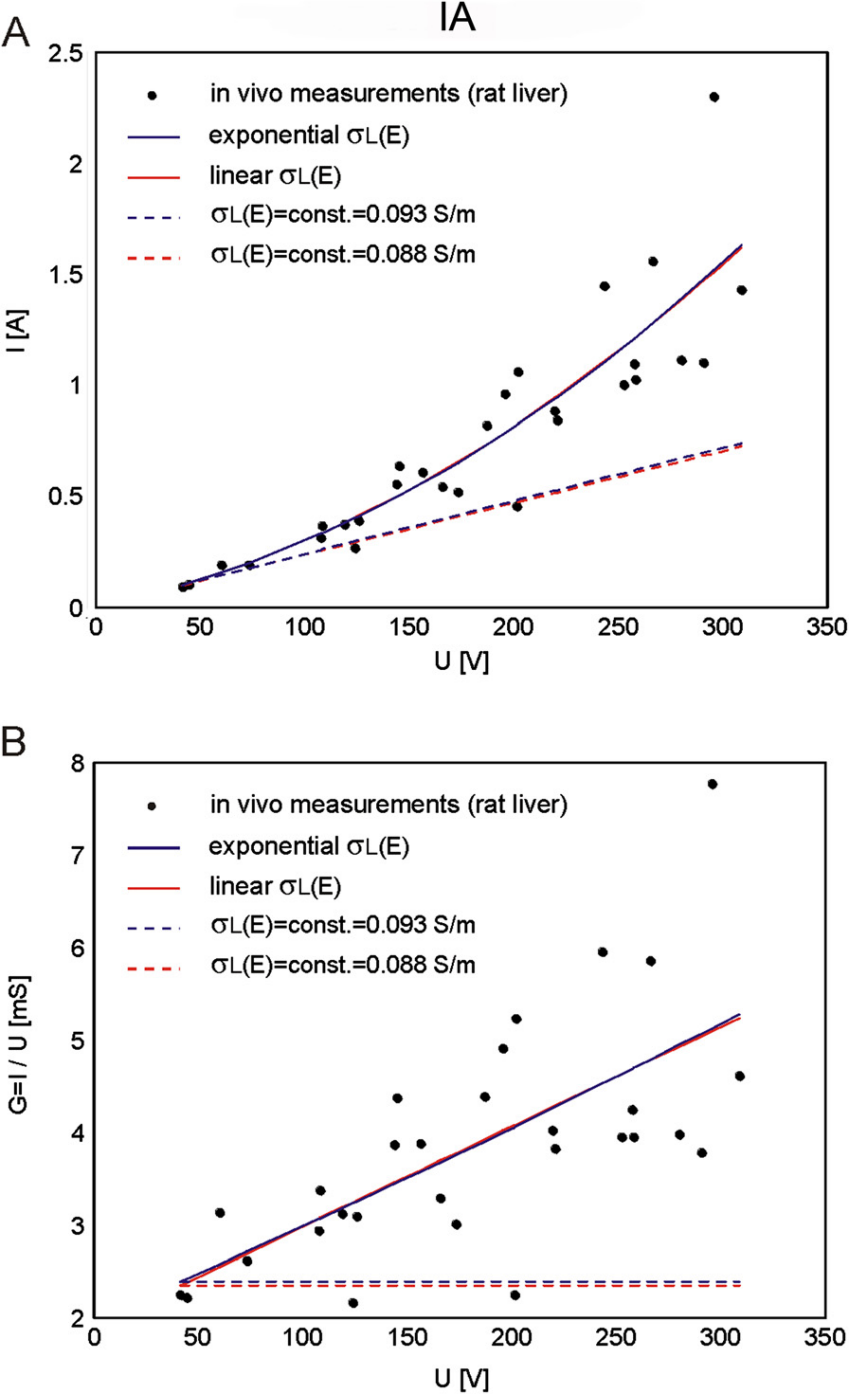
**Comparison of numerical simulations performed with IA to the data measured *in vivo*. A**. Electric current vs. voltage relationships I(U) calculated in nonlinear models (with linear and exponential σ_L_(E)), calculated in linear models (with σ_L_(E) = σ_0_ = const.) and measured *in vivo* and **B**. Conductance vs. voltage relationships G(U) calculated in nonlinear models (with linear and exponential σ_L_(E)), calculated in linear models (with σ_L_(E) = σ_0_ = const.) and measured *in vivo.*

The calculated I(U) and G(U) for both identified relationships σ(E) (linear and exponential) by means of IA are shown in Figure 4. In Figure 4 the I(U) and G(U) characteristics with σ(E) were also compared to the corresponding I(U) and G(U) obtained in the model with constant electric conductivity (i.e. with baseline tissue conductivities: σ = const. = 0.093 S/m and σ = const. = 0.088 S/m).

We then performed numerical modeling with NPA with smoothed Heaviside relationships of σ(E) for two predefined baseline electrical conductivities: 0.126 S/m [67] and 0.091 S/m [68]. Within the smoothed Heaviside relationships of σ(E) the conductivity increase factor was chosen to increase by factor of two and three [62] which is in agreement with previously experimentally observed σ increase at the end of pulse [2] For the reversible and irreversible threshold values we have chosen 200 V/cm and 1000 V/cm, respectively [62]. The examined smoothed Heaviside relationships σ(E) with corresponding baseline conductivities and the conductivity increase factors are shown in Figure 3B.

**Figure 5:**
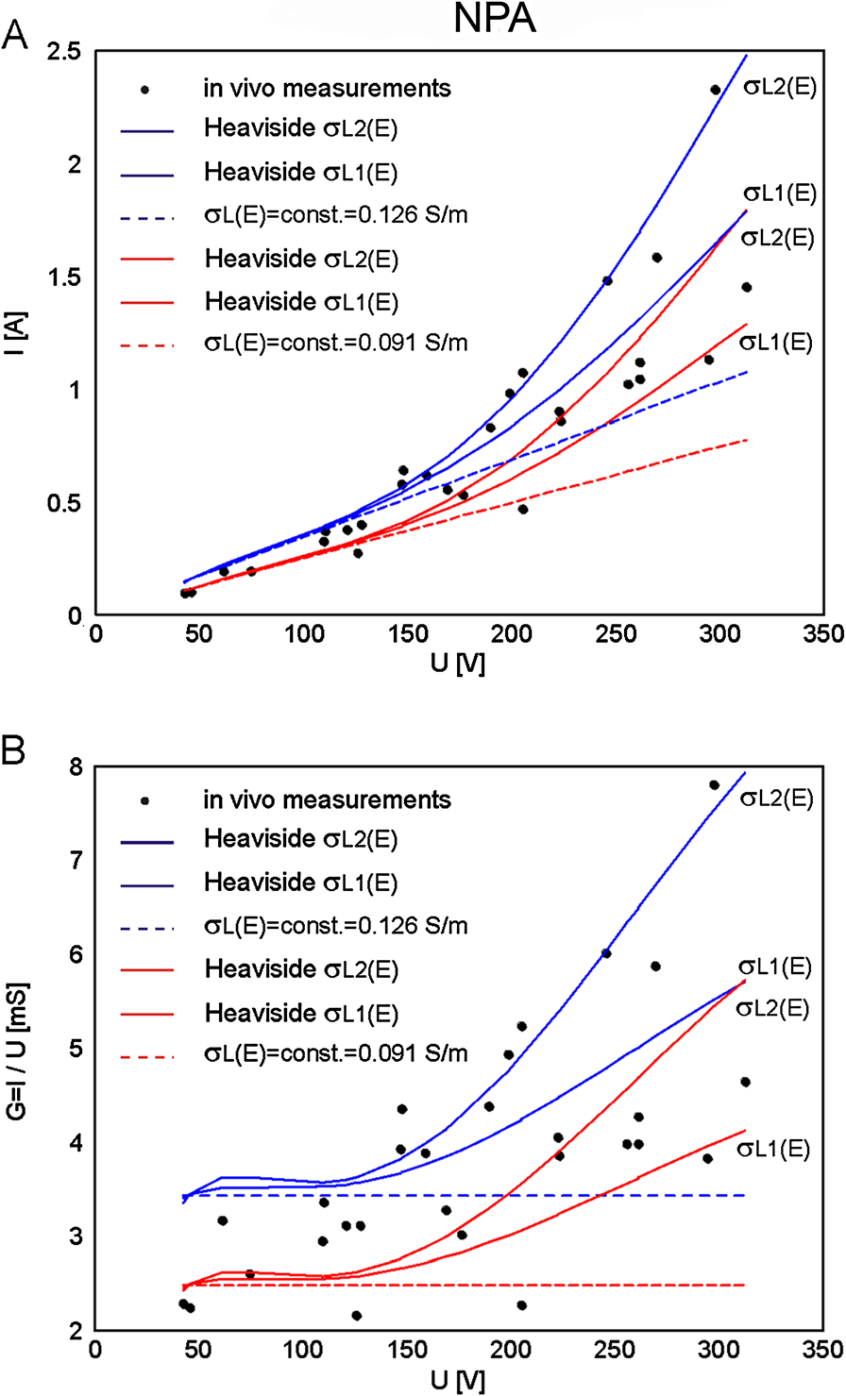
**Comparison of numerical simulations performed with NPA to the data measured *in vivo*. A**. Electric current vs. voltage relationships I(U) calculated in nonlinear models (with linear and exponential σ_L_(E)), calculated in linear models (with σ_L_(E) = σ_0_ = const.) and measured *in vivo* and **B**. Conductance vs. voltage relationships G(U) calculated in nonlinear models (with linear and exponential σ_L_(E)), calculated in linear models (with σ_L_(E) = σ_0_ = const.) and measured *in vivo.*

The obtained I(U) and G(U) characteristics in the models with relationships σ(E) and constant σ (from Figure 3B) are shown in Figure 5A and B, respectively.

Calculated goodness of fit R^2^ measure for the results obtained with IA and NPA are given in Tables 2 and 3, respectively. Almost identical fit was obtained for exponential and linear σ_L_(E) relationship with IA (Table 2). The results of NPA performed in the model with smoothed Heaviside relationship σ_L_(E) and baseline conductivity 0.126 S/m better fitted the experimental data when the conductivity increase factor was chosen to increase by a factor of two - σ_L1_(E), while the model with baseline conductivity 0.093 S/m better fitted the experimental data when the conductivity increase factor was chosen to increase by a factor of three - σ_L2_(E), Table 3.

**Table 2:** **Calculated R**^**2 **^**between IA and *in vivo *data – plate electrodes**

	**Linear model with σ**_**L**_** = const.**	**Nonlinear model with σ**_**L**_**(E)**
	**σ**_**L**_**(0) = 0.093 S/m**	**Exponential σ**_**L**_**(E)**
**R**^**2**^**:**	0.1297	0.7995
	**σ**_**L**_**(0) = 0.088 S/m**	**Linear σ**_**L**_**(E)**
**R**^**2**^**:**	0.1295	0.7993

The calculated goodness-of-fit measure R^2^ between numerical data obtained with linear model (i.e. model with σ_L_ = const.) and nonlinear model (i.e. model with σ_L_(E)) (i.e. model with and *in vivo* data.

**Table 3:** **Calculated R**^**2 **^**between NPA and *in vivo *data – plate electrodes**

	**Linear model with σ**_**L**_** = const.**	**Nonlinear model with smoothed Heaviside σ**_**L**_**(E)**	
	**σ**_**L**_** = 0.126 S/m**	**σ**_**L1**_**(E)**	**σ**_**L2**_**(E)**
**R**^**2**^**:**	0.5637	0.7997	0.4538
	**σ**_**L**_** = 0.091 S/m**	**σ**_**L1**_**(E)**	**σ**_**L2**_**(E)**
**R**^**2**^**:**	0.1296	0.6203	0.7838

The calculated goodness-of-fit measure R^2^ between numerical data obtained with linear model (i.e. model with σ_L_ = const.) and nonlinear model (i.e. model with σ_L_(E)) and *in vivo* data. σ_L1_(E) and σ_L2_(E) stand for the smoothed Heaviside relationships σ_L_(E) with conductivity increase factor (due to electroporatoion) of two and three, respectively.

### Needle electrodes

**Figure 6:**
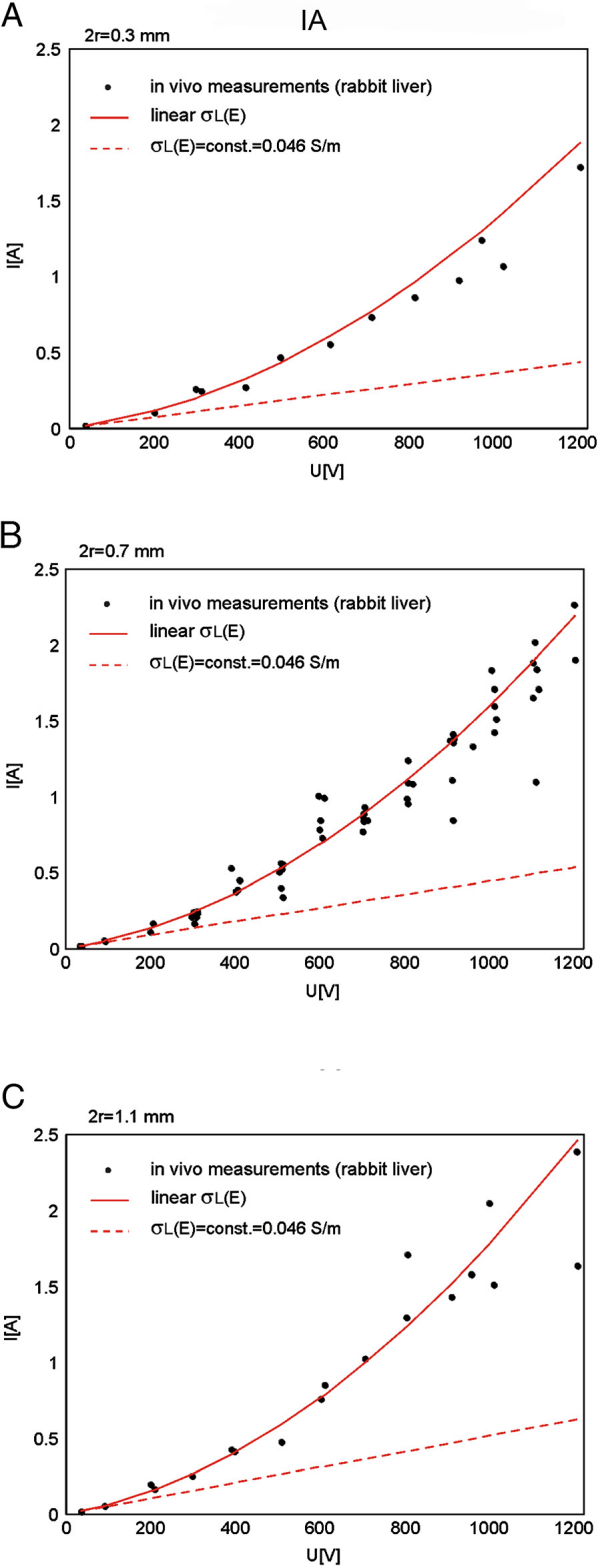
**Comparison of numerical simulations performed by IA to the data measured *in vivo*.** Electric current vs. voltage relationships I(U) calculated in nonlinear models (with linear and exponential σ_L_(E)), calculated in linear models (with σ_L_(E) = σ_0_ = const.) and measured *in vivo* by using needle electrodes with diameters: **A**. 0.3 mm, **B**. 0.7 mm and **C**. 1.1 mm.

**Figure 7:**
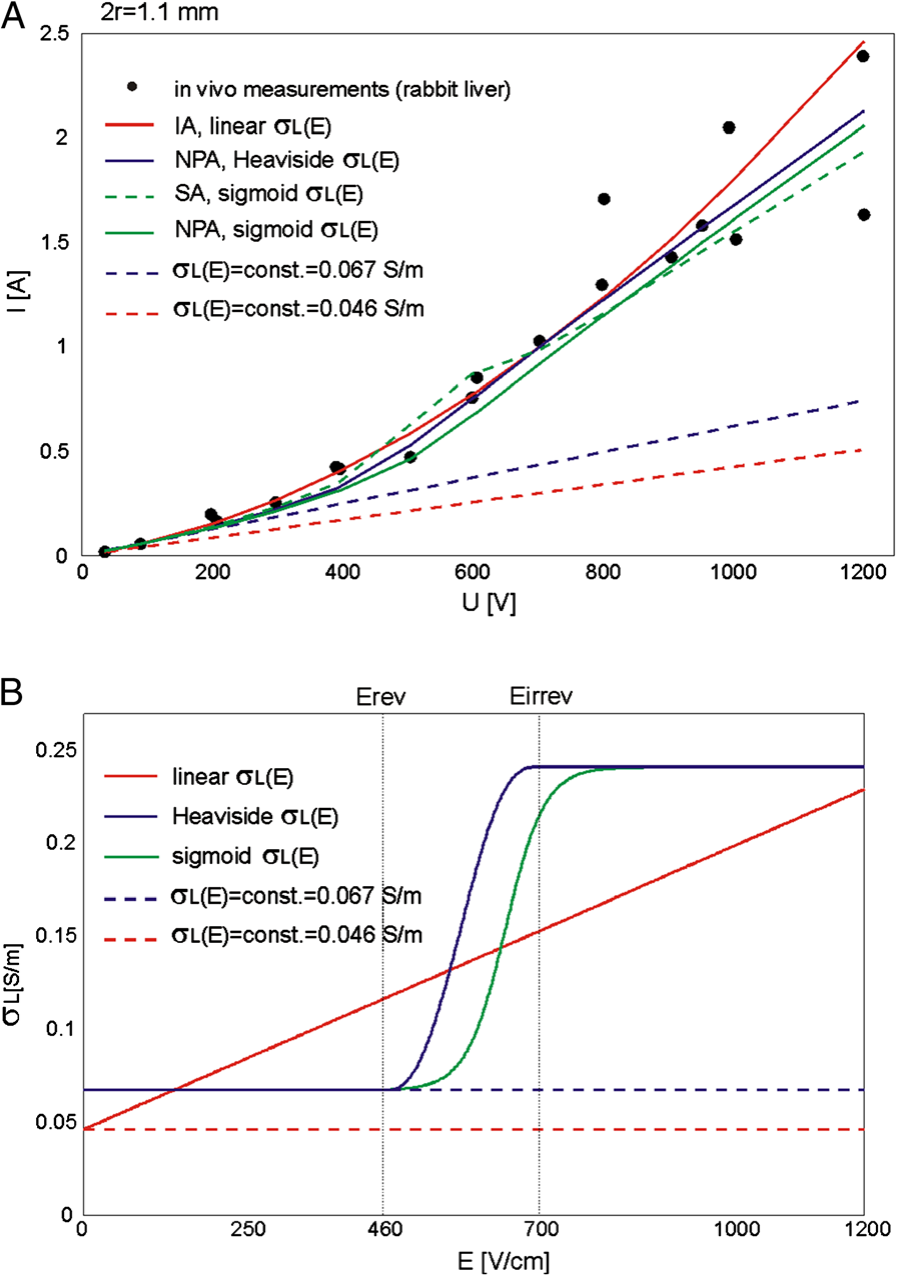
**Comparison of numerical simulations performed by IA, NPA and SA to the data measured *in vivo*. A**. Comparison of U(I) characteristics calculated in nonlinear model and linear to the *in vivo* data. **B**. The relationships σ_L_(E) and constant conductivities σ_L_(E) = σ_0_ = const. in nonlinear and linear models, respectively. The diameter of needle electrodes was 1.1 mm.

In order to identify the baseline liver conductivity and functional dependency of the tissue conductivity on local electric field σ(E) we first performed numerical modeling by using inverse analysis IA on a set of experimental data obtained with needle electrodes with diameter 0.7 mm (Figure 6B). The identified relationship σ(E) was determined to be linear with baseline liver conductivity σ_0_ = 0.046 S/m (Figure 7B). In order to verify the identified parameters of IA we further implemented the same identified relationship on sets of experimental data obtained with needle electrodes with diameters: 0.3 mm, and 1.1 mm, given in Figure 6A and C, respectively and calculated the goodness of fit measure R^2^ for all three models (Table 4). The numerically calculated I(U) relationship taking into account the linear relationship σ(E) was compared to the I(U) with σ = constant corresponding to the identified baseline tissue conductivity (σ = const. = 0.046 S/m) (Figure 6A, B and C).

**Table 4:** **Calculated R**^**2 **^**between IA, NPA and SA and *in vivo *data - needle electrodes**

	**Electrode diameter**		
	**1.1 mm**	**0.7 mm**	**0.3 mm**
**Analysis type with** σ_L_(E)	**R**^**2**^**:**		
IA with linear σ_L_(E)	0.883214	0.875605	0.92873
NPA with smoothed Heaviside σ_L_(E)	0.9192		
NPA with sigmoid σ_L_(E)	0.8964		
SA with sigmoid σ_L_(E):	0.8938		

* The calculated R^2^ for SA is given for the σ_L_(E) with 5 discretization steps. The calculated R^2^ for models with σ being independent of E (σ_L_ = const. = 0.04593 S/m and σ_L_ = const. = 0.067 S/m) was negative.

The calculated goodness-of-fit measure R^2^ between numerical data obtained with nonlinear model (model with σ_L_(E)) and *in vivo* data.

In order to compare the results of NPA and SA to the experimental data we also calculated I(U) relationships for predefined relationships σ(E): smoothed Heaviside with NPA and sigmoid with NPA and SA (Figure 7B). We predefined the baseline liver conductivity, reversible and irreversible threshold values based on previously reported data by Sel and colleagues [58,54]: σ_L0_ = 0.067 S/m, E_rev_ = 460 V/cm and E_irrev_ = 700 V/cm. The NPA and SA were also performed for tissue model with needle electrodes of 1.1 mm diameter given in Figure 1B. Our model corresponds to the one used in [58] in which the SA-analysis was first developed and implemented. The I(U) relationships obtained with σ(E) were compared to the models with constant σ (i.e. σ = 0.067 S/m and σ = 0.046 S/m), Figure 7.

Comparison of U(I) characteristics obtained in the model (needle electrodes, diameter 1.1 mm) with relationships σ(E) and σ = const. to the experimental data by using IA, NPA and SA is shown in Figure 7.

Calculated goodness of fit measure R^2^ for models with needle electrodes obtained with all three different analyses is given in Table 4.

By using SA analysis we calculated the I(U) characteristics for two different discretizations of modeled pulses: 5 steps and 15 steps. In Figure 7 we showed only the I(U) for 5 discretization steps since the goodness-of-fit for I(U) obtained with 15 steps did not significantly differ from the goodness-of-fit obtained with 5 steps: R^2^ = 0.8958 (15 steps) vs. R^2^ = 0.8938 (5 steps), Table 4.

The dashed green curve and the solid green curve in the Figure 7A represent the I(U) relationships obtained with SA and NPA, respectively. In both analyses the same σ(E) was used (i.e. sigmoid function represented with green solid curve in Figure 7B). The difference between the two I(U) relationships occurs due to the different discretisation approaches of σ(E). Namely, the discretisation of σ(E) used in SA is usually coarser (i.e. discretisation of σ(E) is obtained by employing 5 step functions) compared to finer automatic discretization of σ(E) used in NPA.

### Composite tissue

**Figure 8:**
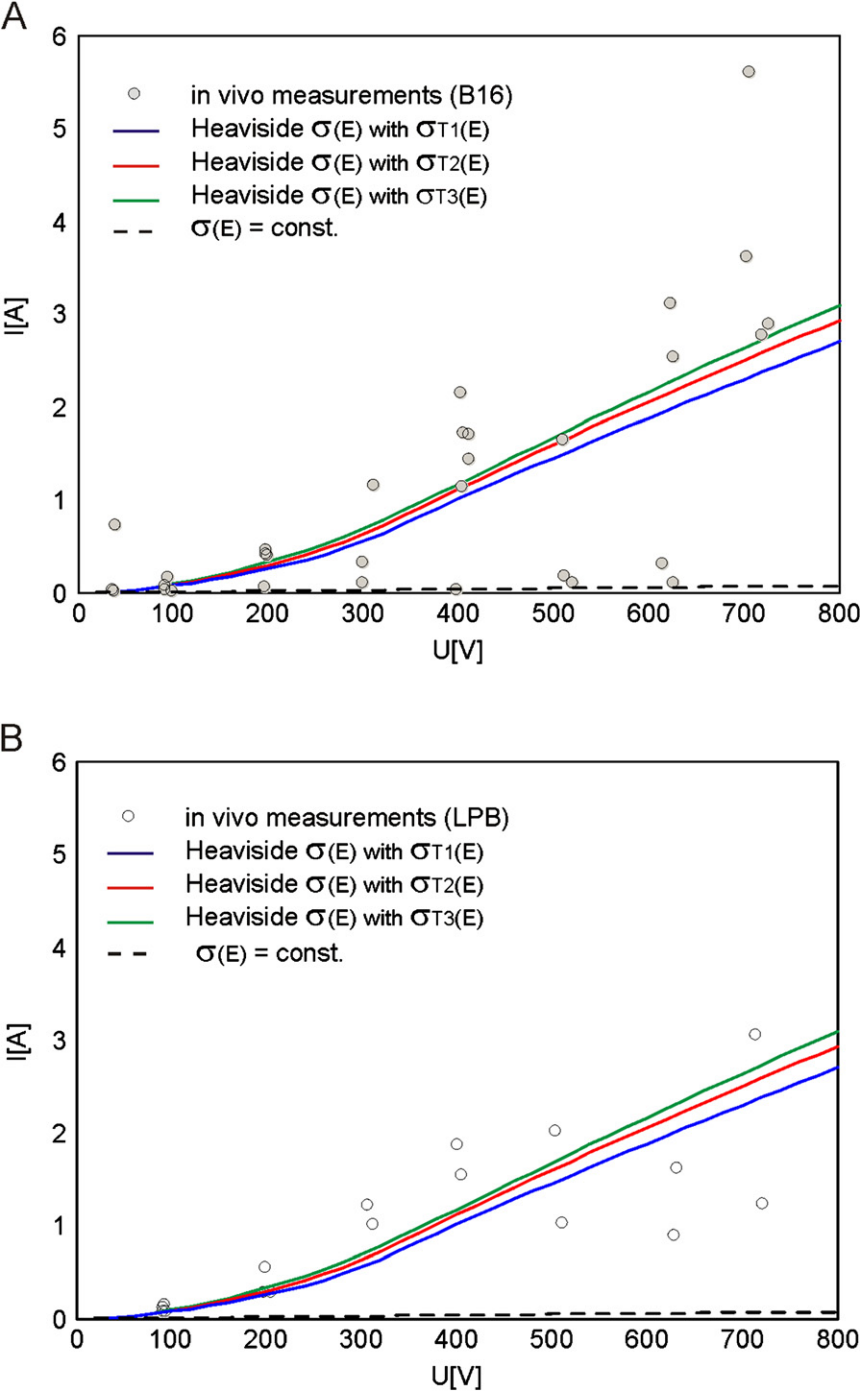
**Numerically calculated vs. *in vivo *measured I(U) relationship in subcutaneous tumors.** Comparison of numerically calculated I [A] (in both nonlinear and linear models) with the experimentally measured I [A] for two types of tumors: **A**. B16 melanoma and **B**. LPB sarcoma. In nonlinear numerical models we examined three different σ_L_(E) of tumors (Table 1 and Figure 9b).

**Figure 9:**
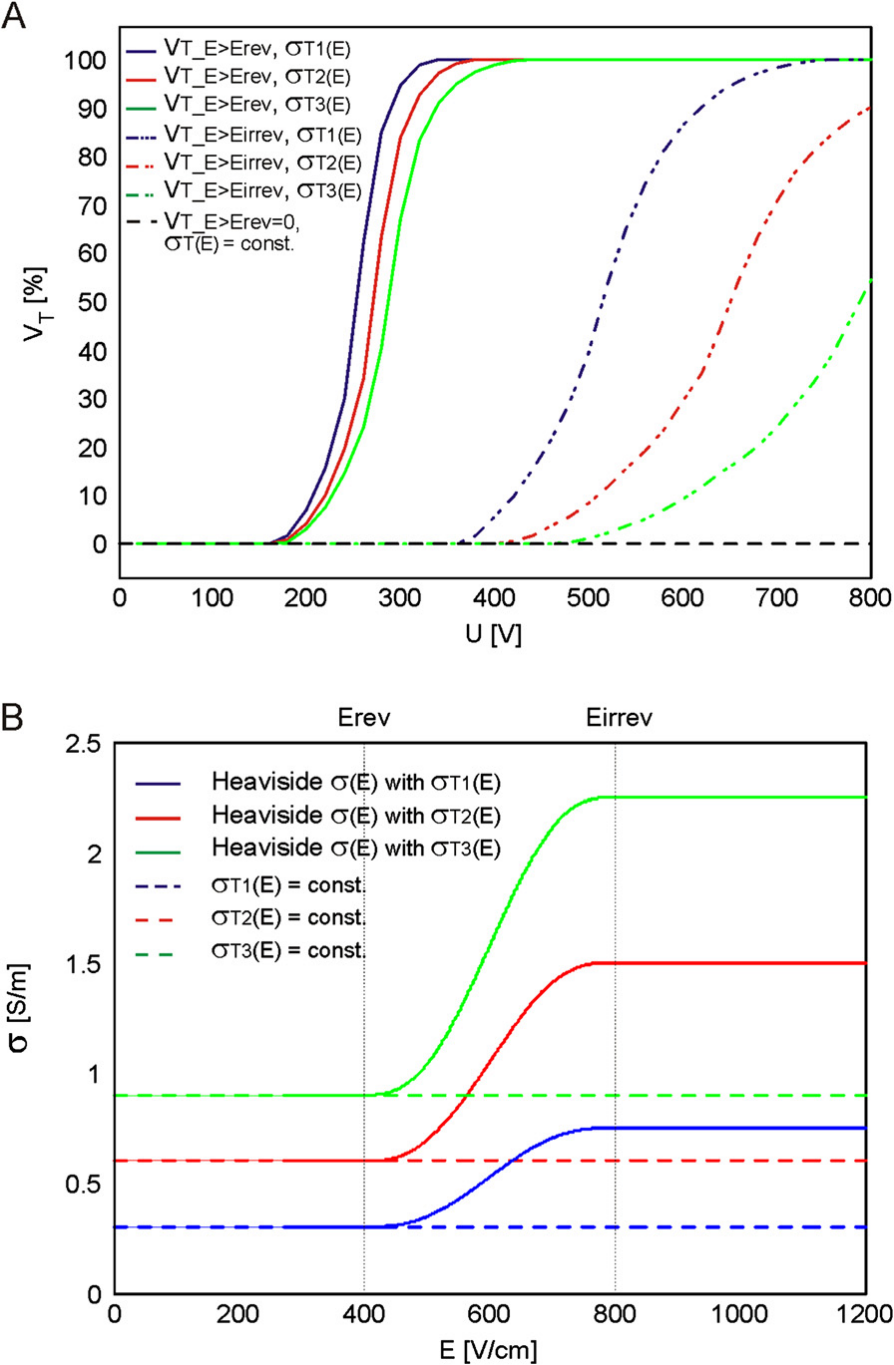
**Reversibly and irreversibly electroporated tumor volume V**_**T **_**[%] in linear (σ**_**T**_**(E) = const.) and nonlinear models (σ**_**T **_**(E)). A**. The calculated percentage of tumor volume V_T_ [%] exposed to the local electric field above E_rev_ and E_irrev_ and **B**. the examined baseline conductivities (σ_T_(E) = const.) and relationships σ_T_(E) of the target tumor tissue.

The comparison of numerically calculated total current I [A] in tumor models (with σ(E) and σ = const. given in Table 1) to the total current measured *in vivo* for two different types of tumors B16 melanoma and LPB sarcoma is given in Figure 8A and B, respectively. The examined σ_T_(E) are shown in Figure 9B.

The calculated goodness of fit R^2^ for two different types of tumors B16 melanoma and LPB sarcoma is given in Table 5.

**Table 5:** **Calculated R**^**2 **^**between model data and *in vivo *data (plate electrodes, NPA)**

	**Tumor B16**	**Tumor LPB**
**Model**	**R**^**2**^**:**	**R**^**2**^**:**
σ_T1_(E)	0.4842	0.5197
σ_T2_(E)	0.4992	0.4799
σ_T3_(E)	0.5028	0.4322

* Subcutaneous tumor models with σ being independent of E (i.e. σ_T_, σ_SE_, σ_DF_, σ_M⊥_, σ_M||_ = const.) have negative R^2^.

The calculated goodness-of-fit measure R^2^ between numerical data obtained with nonlinear model (i.e. model with σ(E)) and *in vivo* data obtained for B16 melanoma and LPB sarcoma.

The calculated volume fraction (i.e. volume percentage V [%]) of the target tumor tissue exposed to the local the electric field distribution above E_rev_ and E_irrev_ for three different σ(E) relationships of target tumor tissue (i.e. σ_T1_(E), σ_T2_(E) and σ_T3_(E)) and for σ independent of E (i.e. σ_T_ = const.), is presented in Figure 9. The entire volume of the target tumor tissue (V_T_E>Erev_ = 100%) was exposed to the local electric field above E_rev_ at different applied voltages for different target tumor tissue conductivities σ_T_(E): U = 340 V (for σ_T1_(E)), U = 400 V (for σ_T2_(E)) and U = 440 V (for σ_T3_(E)). The exposure of the target tumor tissue to the *E* above irreversible threshold value V_T_E > Eirrev_ was also obtained for different U for different σ_T_(E): V_T_E >Eirrev_ = 100% for σ_T1_(E) was obtained at U = 720 V Figure 9A, while the V_T_E >Eirrev_ =100% for σ_T2_(E) and σ_T3_(E) was obtained for U > 800 V (in our study the U was applied from 20 V to 800 V).

The volume of the target tumor tissue V_T_ in the subcutaneous model with constant conductivities of all tissue σ_SE_, σ_DF_, σ_M⊥_ and σ_M||_ and σ_T_ = const. was zero (V_T_E>Erev_ = 0) as shown in Figure 9A.

**Figure 10:**
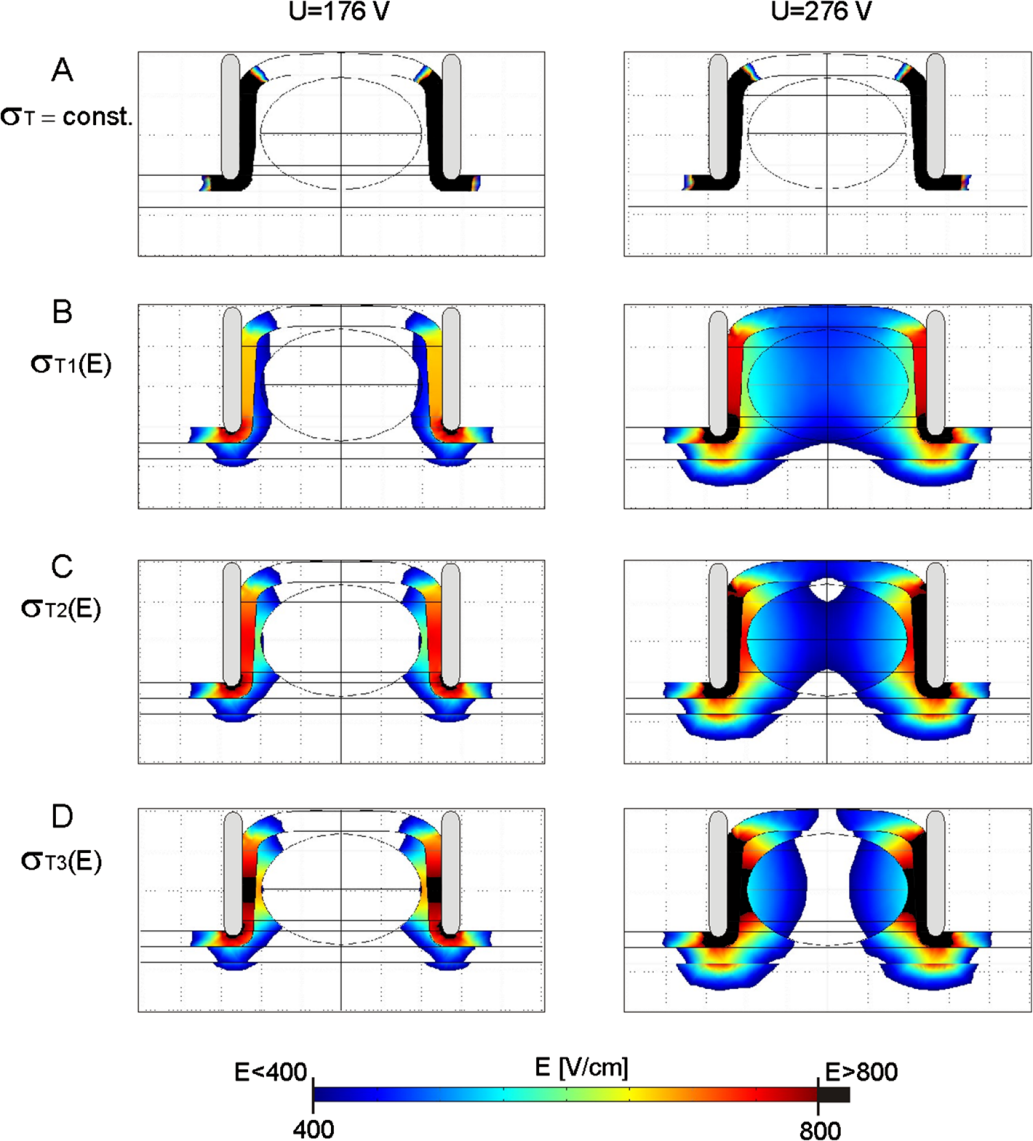
**Local electric field E in the numerical model of subcutaneous tumor.** Calculated and visualized local electric field E distribution for two different applied voltages U = 176 V and U = 276 V in XY cross section plane of the subcutaneous tumor model. The E is calculated for the following σ_T_ of the target tumor: **A**. σ_T_ = const., **B**. σ_T1_(E), **C**. σ_T2_(E) and **D**. σ_T3_(E).

We visualized the calculated local electric field distribution within the model of subcutaneous tumor tissue for two different applied voltages U = 176 V and U = 276 V (Figure 10) in XY cross-section plane (see Figure 2). At the applied voltage U = 176 V the local electric field in the target tumor tissue just started to enter the tumor, while the U = 276 V was chosen to show the difference in distribution of E for different electric properties of the target tumor tissue σ_T_(E).

The electric field distribution is calculated for four different conductivities of target tumor tissue σ_T_: Figure 10A σ_T_ = const., Figure 10B σ_T1_(E), Figure 10C σ_T2_(E) and Figure 10D σ_T3_(E). The electric field distribution in Figure 10 was displayed in the range from the reversible E_rev_ and irreversible threshold value E_irrev_ of the target tumor tissue 400 V/cm ≤ E ≤ 800 V/cm.

## Discussion

In a recent study of robustness of electrochemotherapy treatment planning based on analysis of local electric field distribution E the authors show that the uncertainties in predefined dielectric properties of the treated tissues and in the rate of increase in electric conductivity due to the electroporation have large effect on treatment effectiveness, which indicated that more experimental and numerical research is needed [43]. Numerous other numerical and experimental studies also showed that the electric conductivity changes during tissue electroporation. However, in preclinical studies and clinical studies the electroporated tissues are often considered as conductive materials with constant electric conductivities.

In our study we therefore investigated whether the functional dependency of electric properties and of local electric field distribution within treated tissues need to be taken into account when modeling tissue response to the electroporation pulses.

We compared linear (i.e. tissue conductivity is constant σ = const.) and non-linear model (tissue conductivity depends on local electric field σ(E)) to the experimental data *in vivo *[56]. We built our numerical models so as to fit as accurately as possible the tissue geometry, the electrode geometry and the contact surface between the electrodes and tissue treated in *in vivo* experiments. In the first part of our study we performed the comparison for single tissue (i.e. one type of tissue) for two types of electrodes: plate and needle electrodes. For single tissue modeling we analyzed the experimental data performed in rat liver treated with plate electrodes and in rabbit liver treated with needle electrodes (Figure 1).

The main findings of the single tissue analysis were then applied to the model of composite tissue (i.e. the tissue composed of different types of tissues with different electric conductivities). The study of composite tissue was performed for subcutaneous tumor that consisted of stratum corneum, dermis, epidermis, connective and fatty tissue, tumor tissue and muscle tissue and was treated with plate electrodes (Figure 2).

Numerical simulations in our study were performed by using three different modeling approaches: IA [60,61], NPA [62] and SA [58].

In our study the IA was first applied for numerical modeling of electroporation in a single tissue (i.e. rat liver tissue) treated with plate electrodes. The relationship between local electric field distribution and the conductivity of liver tissue σ(E) was described by linear (Eq. 7) and exponential functions (Eq. 8). The parameters of these two functions (parameters σ_0_ and k_σ_ of linear function and parameters σ_0_, c_1_ and c_2_ of exponential function) were than calculated with IA-analysis algorithm by fitting the data of numerically calculated to the experimentally measured values of total electric current flowing through the tissue (Figure 3). The parameters σ_0_ in both functions σ(E) (linear and exponential) represented the baseline tissue conductivities for the condition σ_0_ = σ(0)) (i.e. the initial conductivity of non-electroporated tissue). We calculated these parameters to be σ_0_ = 0.088145 S/m (linear function Eq. 7) and σ_0_ = 0.092768 S/m (exponential function Eq. 8). The numerically calculated values of σ_0_ of both functions correspond well to the measured conductivities of rat liver tissue published by Gabriel and colleagues [68]. These results show that the IA allows for identification of the initial tissue conductivity based on measured values of total electric current flowing through the tissue exposed to the electroporation pulses. In order to compare the numerical models of liver tissue that consider exponential and linear σ(E) relationship to the numerical model with constant conductivity (σ(E) = const.) we used the calculated data σ_0_ for development of the models with constant conductivities: σ(E) = σ_0_ = const. The calculation of goodness of fit measure (R^2^) of the numerical models that take into account the relationship σ(E) fit better experimental data compared to the models with constant conductivity: linear σ(E) R^2^ = 0.7993 and exponential σ(E) R^2^ = 0.7995 vs. σ(E) = σ_0_ = 0.088145 S/m = const. R^2^ = 0.1295 and σ(E) = σ_0_ = 0.092768 S/m = const. R^2^ = 0.1297 (Table 2)). These results show that the IA also allows for identification of functional dependency of tissue conductivity σ(E) of the electroporated tissue. The results of our study thus demonstrate that by using IA both the baseline electric conductivity for non-electroporated tissue (for the condition σ(0) = const. for E = 0 or E < E_rev_) and the σ(E) relationship of the tissue exposed to U that results in tissue electroporation (E > E_rev_) can be identified.

The calculation of tissue conductance G [S] show that the conductance of the models with σ(E) increased with increase of the applied voltage following the measured data of G Figure 3D. We can observe that the models G = const. do not describe the electroporation process of the tissue which is in agreement with our previous findings [2,56], as well as recent multiscale modeling [57].

In order to compare numerical simulations obtained by nonlinear parametric analysis (NPA) to the numerical simulations obtained with inverse analysis (IA) we used the same geometry of liver tissue and electrodes (Figure 1A) and electric properties of tissue (σ_L0_ = const. and σ(E)) as used in our previous work [62]. Namely, for the initial parameters of tissue conductivity we used two different measured values from the literature σ_L0_ = 0.091 S/m (pig liver) [68] and σ_L0_ = 0.124 S/m (rat liver) [67]. The relationship between the local electric field and tissue conductivity σ(E) was implemented to the tissue model as smoothed Heaviside’s function. The numerical calculations were performed for both constant conductivity σ = const. and σ(E) with conductivity increase factor of two and three (Figure 3B), which is in agreement with experimentally measured conductivity increase at the end of the pulse [2,58]. Our results show that the numerical models with σ(E) better fit experimental data than models with σ = const. (Table 3). Numerical model with initial conductivity of rat liver better fit experimental data for the conductivity increase factor of two (R^2^ = 0.7997), the numerical model with initial conductivity of pig liver better fit experimental data for the conductivity increase factor of three (R^2^ = 0.7838). When comparing the numerical results obtained with NPA to the results obtained with IA we can conclude that similar results and good agreement between numerical and experimental data can be obtained with both modeling approaches when σ(E) relationship is taken into account (regardless of the shape of the σ(E)). Both approaches show that the models with σ(E) fit the measured data considerably better than models with constant conductivity. However, more precise measurements are needed for determination of the shape of the σ(E) function that might be tissue type specific.

In the second part of our study we built numerical models of single tissue (rabbit liver) treated with needle electrodes of three different diameters 0.3 mm, 0.7 mm and 1.1, that were used also in *in vivo* experiments. By using IA we first identified the σ(E) for the tissue model with needle electrodes with diameter of 0.7 mm, as in this case we analyzed the maximum number of measurements Figure 6. We found that the best fit of numerical data with experimental data was obtained with linear σ(E) function (Figure 6B) with R^2^ = 0.875605 (Table 3). The identified value of the initial tissue conductivity was σ_0_ = 0.04593 S/m which correspond to the measured conductivity of rabbit liver (summarized in [58]). The identified linear function in the model with 0.7 mm was then applied to the models with 0.3 mm and 1.1 mm and good agreement between calculated data were obtained R^2^ = 0.92873 (for 0.3 mm) and R^2^ = 0.883214 (for 1.1 mm) (Table 4).

For the model with electrode diameter 1.1 mm we also performed NPA with smoothed Heaviside and sigmoid σ (E) function and sequential analysis with sigmoid function σ(E) with parameters determined by [58]. We also performed numerical analysis with σ(E) = const. The comparison of the model with σ(E) = const. to the numerical models with σ(E) showed that models with σ(E) fit experimental data considerably better then models with σ(E) = const. (Table 4). When comparing numerical results of different modeling approaches (Figure 7) we further observed that the best fit between the numerical and experimental data was obtained with NPA with smoothed Heaviside’s function R^2^ = 0.9192 (Table 4).

Based on the comparison of all three modeling approaches validated with experimental measurements we determined the important advantages for electroporation process modeling of each of the approaches. Namely, the IA can be used as a modeling approach for the situations when the conductivity of treated tissue is difficult to be measured before or during electroporation. The NPA allows for parametric analysis of electric current at the end of the applied voltage pulses while the SA allows also an insight into the electroporation process (i.e. the course of σ(E) changes) during the applied pulse. Although, the NPA and SA modeling approaches require the baseline conductivity and the σ(E) need to be predefined, the important advantage is that these two modeling approaches are relatively simple to be used in commercial software which is accessible and widely used.

In the third part of our study we built the numerical model of subcutaneous tumor in order to examine the influence of tissue heterogeneity on the electric field distribution during electroporation of a composite tissue (i.e. tumor model composed of different types of tissues). We compared the numerical calculation of total electric current in the tumor model with constant conductivity of all constituent tissues and the numerical calculation of total electric current in the tumor model that took into account σ(E) of all involved tissues to the experimental measurements *in vivo* carried out in two different types of tumors: B16 melanoma (Figure 8A) and LPB sarcoma (Figure 8B). For all tissues in the model we implemented smoothed Heaviside’s function σ(E).

Based on the comparison of the numerical and experimental data *in vivo* we demonstrated that the model that took into account σ(E) of all involved tissues fits better *in vivo* data than model with constant conductivities (Table 5). Our results further show that the subcutaneous tumor model with higher conductivity of target tumor tissue σ_T3_(E) fits better the *in vivo* data of B16 melanoma while the model with σ_T1_(E) better fits the *in vivo* data of LPB sarcoma subcutaneous tumor (Table 5), suggesting that B16 melanoma has higher conductivity than LBP sarcoma. The visualization of local E in the subcutaneous tumor model demonstrates that the more conductive the target tumor tissue the higher the intensity of E in the surrounding tissue and the lower E in the tumor tissue (Figure 10A and B), which is in agreement with our previous findings [70].

We also calculated the tumor volume exposed to the local electric field above E_rev_ and E_irrev_ (Figure 9A) and demonstrated that more conductive target tumor tissue were more difficult to be successfully electroporated, since they require higher voltage U to be applied to the electrodes in order to expose the entire volume of the target tumor above the E_rev_ or higher, which also results in higher intensity of E in the skin and muscle and subsequently may induce more damage to the surrounding healthy tissue (Figure 10). We also demonstrated that the electroporation of more conductive tissues resulted in higher values of total electric current I [A] flowing through tissue (Figure 8). The exposure of the target tumor tissue to the E above irreversible threshold value also depends on the electric properties of the target tumor tissue σ_T_(E). It needs to be emphasized that the subcutaneous model with constant conductivities of all tissues (i.e. in the subcutaneous tumor model that do not take into account the relationship σ(E)) showed that a very high electric field (E > E_irrev_) was concentrated only in the stratum corneum while the target tumor tissue was not treated (Figure 10A). Furthermore, the volume of the target tumor tissue V_T_ exposed to the E > E_rev_ in the subcutaneous model with constant conductivities of all tissue was zero (Figure 9A).

## Conclusions

In our study we investigated whether the increase in electric conductivity of tissues needs to be taken into account when modeling tissue response to the electroporation pulses and how it affects the local electric distribution within electroporated tissues. We examined electric field distribution during electroporation in both linear models (i.e. tissue conductivity is constant) and non-linear models (i.e. tissue conductivity is electric field dependent). The results show that nonlinear models fit experimental data better than linear models. We demonstrated that the tissue conductivity as a function of electric field (i.e. σ(E)) needs to be considered when modeling tissue behavior during electroporation. Therefore, the σ(E) relationship needs to be taken into account when an electroporation based treatment is planned or investigated. The findings of our study can be of great importance for precise treatment planning of electroporation based therapies and treatments (e.g. for electrochemotherapy, gene electrotransfer for gene therapy and DNA vaccination, tissue ablation with irreversible electroporation and transdermal drug delivery). Particularly, for an electroporation based therapy of deep-seated cutaneous tumors and tumors in internal organs, such as for example bone, brain or muscle tissue that are surrounded by other tissues having different electric properties an individualized patient-specific treatment planning that takes into account heterogeneity of electric conductivity and local electric field distribution within all exposed tissues is required.

## Competing interests

The authors declare that they have no competing interests.

## Authors’ contributions

SC and DM designed the study. PS, TS and TR performed modeling with IA. IL and SC performed modeling with NPA. SC performed modeling with SA. All authors were involved in the analysis of numerical and experimental data. All authors were involved in the preparing of the manuscript. All authors read and approved the final manuscript.
